# Disease mechanism and novel drug therapies for atrial fibrillation

**DOI:** 10.1515/medgen-2025-2005

**Published:** 2025-04-08

**Authors:** Felix Wiedmann, Constanze Schmidt

**Affiliations:** Medical University Hospital Heidelberg Department of Cardiology Im Neuenheimer Feld 410 69120 Heidelberg Germany; Medical University Hospital Heidelberg Department of Cardiology Im Neuenheimer Feld 410 69120 Heidelberg Germany

**Keywords:** Atrial fibrillation, TASK-1 potassium channels, cellular electrophysiology, Antiarrhythmic therapy, Molecular pathophysiology

## Abstract

Atrial fibrillation (AF), the most common sustained cardiac arrhythmia, affects over 3 % of adults globally, increasing risks for stroke, heart failure, and cognitive decline. Early rhythm control shows promise in improving AF prognosis, and catheter ablation remains an effective, safe option, especially for paroxysmal AF. However, high recurrence rates with antiarrhythmic drugs and ablation persist, particularly in cases of persistent AF. Emerging research on molecular mechanisms has led to innovative therapeutic strategies targeting these pathways, offering hope for more effective AF management. This review explores recent insights into the complex pathophysiology of AF, with a particular focus on ion channel dysfunction, calcium mishandling, oxidative stress, and fibrosis. It further considers how these factors will inspire new therapeutic options.

## Abbreviations

AAD, antiarrhythmic drug; AF, atrial fibrillation; APD, atrial action potential duration; cAF, chronic atrial fibrillation; DADs, delayed afterdepolarizations; EADs, early afterdepolarizations; EMA, European Medicines Agency; ESC, European Society of Cardiology; FDA, Food and Drug Administration; HFrEF, heart failure with reduced ejection fraction; *I*_K,ACh_, acetylcholine-activated K^+^ current; *I*_K1_, inward rectifier potassium currents; *I*_Ks_, slow delayed rectifier potassium current; *I*_Kur_, ultra-rapid delayed rectifier potassium current; IL-6, interleukin-6; *I*_to_, transient outward K^+^ current; NCX, Na^+^/Ca^2+^ exchanger; ROS, reactive oxygen species; RYR2, ryanodine receptor; SK, small-conductance calcium-dependent potassium channel; SR, sarcoplasmic reticulum; TGF-β, transforming growth factor-beta; TNF-α, tumor necrosis factor-alpha.

## Introduction

Atrial fibrillation (AF) is the most prevalent sustained cardiac arrhythmia globally, affecting an estimated 3–4 % of adults and contributing significantly to morbidity and mortality due to its associations with stroke, heart failure, and cognitive decline [1]. Despite the high burden of AF on healthcare systems worldwide, the pathophysiology underpinning this complex condition remains incompletely understood. Electrophysiological changes, including ion channel dysfunction, calcium mishandling, and atrial fibrosis, contribute to AF initiation and progression [2, 3]. Traditional treatments have largely focused on rate and rhythm control, including anti-arrhythmic drugs and catheter ablation, yet recurrence rates remain high, particularly in patients with persistent AF. Recent advances in the understanding of the molecular mechanisms underlying AF – including disturbances in ion channel function, calcium handling, tissue fibrosis, oxidative stress and alterations of the autonomic nervous system – have yielded new therapeutic targets aimed to address the key cellular and molecular processes underlying AF [4]. This review examines recent advances in the understanding of AF pathophysiology and highlights innovative drug therapies that promise to address underlying disease mechanisms, potentially improving long-term outcomes in AF management.

## Pathophysiology of atrial fibrillation

The pathogenesis of AF involves a complex interplay of molecular and cellular abnormalities that collectively alter atrial structure and function, finally leading to arrhythmogenesis [2, 5].

Electrical remodelling is largely attributed to alterations in atrial ion currents. Potassium channels, specifically, undergo significant dysregulation, leading to shortened atrial action potentials and a reduced refractory period that enhances re-entry susceptibility. Key channels involved include the acetylcholine-activated K^+^ current (*I*_K,ACh_), which becomes constitutively active [6–8], and inward rectifier currents (*I*_K1_) [9–11], both of which contribute to a hyperpolarized state and sustained K^+^ current. Additionally, transient outward K^+^ current (*I*_to_) are downregulated [12], further leading to shortening and the triangulation of atrial action potentials, characteristic for AF. Evidence for dysregulation of the ultra-rapid delayed rectifier (*I*_Kur_) is inconsistent, showing either unchanged or reduced *I*_Kur_ function in chronic AF (cAF) patients [13, 14]. Further, atrial remodeling has been demonstrated to augment the amplitude and density of the slow delayed rectifier potassium current (*I*_Ks_) [15]. It is hypothesized that this phenomenon contributes further to action potential duration (APD) shortening due to the slow deactivation of *I*_Ks_ and the subsequent accumulation at high frequencies [15]. Frequency-dependent *I*_Ks_ inhibition, such as that induced by propafenone, could therefore be a valuable antiarrhythmic strategy, positioning *I*_Ks_ as a potential therapeutic target [15].

Small-conductance calcium-dependent potassium (SK) channels are expressed ubiquitously across the heart. However, their functional impact appears to be more specific to atrial repolarization [16]. Recent studies have shown that certain members of the SK-channel family are upregulated in AF and contribute to pathological APD shortening. This suggests that pharmacological blockade of SK channels could be a new approach for developing rhythm control therapy [16–19].

Additionally, TASK-1, a background two-pore-domain potassium (K_2P_) channel, which displays atrial specific expression in the human heart and has gained attention for its role in AF due to its influence on atrial APD [20–22]. TASK-1 potassium currents have recently been described to be upregulated in AF, contributing to pathological APD shortening and facilitating a substrate for AF maintenance [20, 23, 24]. Given these findings, TASK-1 presents a promising therapeutic target. Pharmacological inhibition of TASK-1, using gene therapy approaches or experimental pharmacological inhibitors like A293, has demonstrated potential in preclinical models to restore normal APD and reduce AF incidence without affecting ventricular electrophysiology, offering a pathway for atrial-selective antiarrhythmic therapy [25–28].

Calcium (Ca^2+^) handling abnormalities also play a crucial role in AF pathophysiology [3]. In AF, there is a marked reduction in the L-type Ca^2+^ current (*I*_CaL_), partly due to downregulation of the Ca_V_1.2 subunit as well as respective beta-subunits and functional modifications by kinases and phosphatases that reduce the open probability of these channels [29]. Disruptions in calcium cycling within atrial myocytes promote ectopic firing and triggered activity. For instance, enhanced sarcoplasmic reticulum (SR) Ca^2+^ leak due to ryanodine receptor (RYR2) dysfunction leads to increased cytosolic Ca^2+^ levels, triggering depolarizing currents through the Na^+^/Ca^2+^ exchanger (NCX) and setting off early and delayed afterdepolarizations (EADs and DADs), which act as proarrhythmogenic triggers [30, 31]. Furthermore, upregulation of PDE8B isoforms in cAF was recently identified to contribute to the proarrhythmic reduction of *I*_CaL_ in cAF [32]. Finally, Ca²⁺ handling abnormalities like increased SR Ca²⁺ load and increased RyR2 single-channel open probability may serve as a critical link between the pathophysiology of heart failure with reduced ejection fraction (HFrEF) and AF, two clinically closely connected disease entities [33].

Structural remodelling in AF is characterized by interstitial fibrosis, a process mediated by myofibroblast activation and extracellular matrix deposition. Fibroblasts, which normally provide structural support, are activated under pathological conditions by cytokines and signalling molecules such as transforming growth factor-beta (TGF-β) and angiotensin II [34]. These molecules stimulate fibroblast differentiation into myofibroblasts, leading to excess collagen deposition, atrial wall stiffness, and reduced conduction velocity. The presence of fibrosis creates areas of anisotropic conduction, promoting re-entry circuits and therefore sustaining arrhythmogenic potential [2].

Additionally, oxidative stress and inflammatory signalling are integral to AF development and progression. Elevated production of reactive oxygen species (ROS) in AF promotes oxidative damage contributing to atrial remodelling. ROS generation is often linked to the activity of NADPH oxidase and mitochondrial dysfunction, which impair cellular electrophysiology and calcium handling [35]. Concurrently, pro-inflammatory cytokines, including interleukin-6 (IL-6) and tumor necrosis factor-alpha (TNF-α), promote myocyte apoptosis and extracellular matrix remodelling. This inflammation-driven fibrosis further destabilizes atrial tissue conductivity, sustaining the arrhythmogenic substrate [36].

Changes in autonomic tone also contribute to AF susceptibility. Increased sympathetic and parasympathetic activity destabilizes the atrial substrate, promoting trigger activity and re-entry. Vagal stimulation, for example, can enhance *I*_K,ACh_ currents, shortening the APD and fostering onset of AF episodes [37]. Sympathetic overactivity elevates intracellular Ca^2+^ via beta-adrenergic signalling, leading to abnormal automaticity and increased ectopic triggers [38].

Taken together, the molecular pathophysiology of AF underscores a multi-factorial disorder in which electrical, structural, and autonomic mechanisms interact to perpetuate arrhythmogenesis. This evolving understanding has led to therapeutic interest in specifically targeting the molecular underpinnings of distinct AF subtypes.

## Genetic predisposition

Genetic predisposition plays a significant role in the development and progression of AF, with numerous studies identifying both common genetic variants and rare mutations that contribute to AF susceptibility. Heritable factors have been implicated in around 20–30 % of AF cases, highlighting the importance of specific genetic profiles in atrial electrophysiology and structure [39]. The most studied genetic variations include those affecting cardiac structural proteins, ion channels, and atrial-specific regulatory genes [40].

Genetic mutations affecting cardiac structural proteins contribute to AF through mechanisms involving atrial dilation, fibrosis, and remodelling. Mutations in *MYL4* (encoding myosin light-chain 4), *MYH7* (encoding β-myosin heavy chain) and *LMNA* (lamin A/C) are associated with increased atrial fibrosis and dilatation, which destabilizes atrial electrophysiology and increases the likelihood of arrhythmic activity [41, 42]. Additionally, *TTN* (titin) mutations, which are also commonly associated with dilated cardiomyopathy, increase the risk of AF by contributing to atrial wall thinning and reducing contractile function, facilitating arrhythmogenic re-entry pathways [40].

Loss-of-function mutations in *SCN5A*, a gene encoding the cardiac Na^+^ channel Na_V_1.5, have been linked to reduced excitability and conduction velocity, promoting re-entrant arrhythmias. Further, both gain-of-function and loss-of-function mutations in the *KCNQ1* gene which encodes the alpha subunit *I*_Ks_ were linked to early onset AF. Finally, rare variants in the atrial-specific ion-channel gene *KCNA5* have been reported to co-segregate with early-onset AF in a limited number of families. Although numerous other ion-channel genes have been suggested as potential contributors to monogenic AF, these findings are often based on studies involving small families or candidate gene approaches, with limited replication in larger cohorts. In fact, recent evidence indicates that the prevalence of potentially causative variants in arrhythmia-related genes apart from *SCN5A* and *KCNQ1* remains low in cases of early-onset AF [40].

Genes involved in atrial development and atrial-specific signalling pathways also predispose individuals to AF. One of the most notable examples is *PITX2*, a transcription factor gene crucial for left-right cardiac asymmetry and pulmonary vein myocardial sleeve development. *PITX2* mutations and decreased expression are associated with a higher incidence of AF, likely due to abnormal pulmonary vein ectopy and atrial conduction abnormalities. Another gene, *NKX2-5*, has been implicated in familial AF cases due to its role in cardiac development and the structural integrity of the atria [39, 43, 44].

Advances in genome-wide association studies have identified multiple common genetic loci associated with AF, such as those on chromosome 4q25 near the *PITX2* gene, as well as loci on chromosomes 16q22 and 1q21.1 [45]. Such loci are not directly linked to specific pathogenic mutations but rather involve single nucleotide polymorphisms (SNPs) that appear to increase AF risk by influencing gene expression, atrial size, and electrical activity. These findings underscore a polygenic component to AF, where multiple small-effect genetic variants collectively increase AF susceptibility. They further open the possibility of developing polygenic risk scores to predict individual susceptibility to AF based on the cumulative effect of identified SNPs [46].

Identification of AF-linked genetic mutations and variants offers promising avenues for precision medicine. The 2023 American College of Cardiology/American Heart Association guidelines for AF provide a Class IIb recommendation for genetic testing in individuals under 45 years of age with AF and no apparent risk factors [47]. The yield of rare pathogenic variants in early-onset AF patients ranges from approximately 4 % to 11 %, comparable to that in non-familial dilated cardiomyopathy [40]. Genetic screening for high-risk individuals may aid in early intervention and risk factor modification, potentially delaying AF onset. Finally, significant clinical and genetic overlap exists between AF, heritable ventricular cardiomyopathies, and arrhythmia syndromes, suggesting that AF may serve as an early indicator of severe ventricular disease [40].

## Current treatment landscape

The 2024 European Society of Cardiology (ESC)-endorsed guidelines for AF recommend treatment in accordance with the AF-CARE pathway, which comprises the following four elements: [C] Comorbidity and risk factor management; [A] Avoidance of stroke and thromboembolism; [R] Symptom reduction through rate and rhythm control; and [E] Evaluation with ongoing reassessment [1].

The pharmacological management of AF includes rate control, which focuses on controlling the heart rate to alleviate symptoms, and rhythm control, which aims to maintain sinus rhythm, reduce AF episodes, and improve quality of life, particularly for patients where ablation is unsuitable or declined. Tailoring antiarrhythmic drug (AAD) choices to patient-specific factors, such as underlying heart disease, comorbidities, and prior responses to medication is of utmost importance. For instance, flecainide and propafenone are recommended for cardioversion in patients without structural heart disease due to their rapid efficacy, but are contraindicated for patients with severe ventricular hypertrophy, coronary artery disease, or Brugada syndrome [1].

Additionally, the “pill-in-the-pocket” approach using a single oral dose of flecainide or propafenone is recommended for selected patients with infrequent, symptomatic paroxysmal AF, allowing for self-administration after appropriate assessment and in-hospital validation to ensure safety and efficacy [1].

The class III “multichannel blocker” amiodarone constitutes a versatile antiarrhythmic drug with unique properties encompassing all four Vaughan-Williams classes, making it effective for managing both atrial and ventricular arrhythmias [48]. Its use is particularly advantageous in patients with heart failure or left ventricular dysfunction due to its minimal proarrhythmic risk [1]. However, its clinical application is limited by significant extracardiac toxicities, drug interactions, and the need for regular monitoring. Despite these challenges, amiodarone remains an essential option in arrhythmia management when carefully administered [1, 48]. While AADs do not entirely eliminate AF recurrence, their usage can decrease episode frequency and symptom severity, contributing to the patient-centred objective of better overall disease management.

Catheter ablation is widely recognized as an effective treatment for patients with AF, especially for those who are unresponsive or intolerant to AADs [49, 50]. According to the ESC 2024 guidelines, catheter ablation is recommended as a first-line rhythm control strategy for patients with paroxysmal AF and is suggested for patients with persistent AF when other therapeutic options fail [1]. This approach offers significant symptomatic relief and reduces the frequency and severity of AF episodes by electrically isolating the pulmonary veins, which are often responsible for AF triggers. Pulmonary vein isolation (PVI) is currently the foundational technique for AF ablation, although there is ongoing research to optimize strategies for patients with non-paroxysmal AF and for individuals with comorbidities such as heart failure [47].

In patients with HFrEF, catheter ablation has demonstrated promising benefits, including reduced hospitalizations, improvement in left ventricular function, and overall enhanced survival in some studies. For these patients, ablation can reverse tachycardia-induced cardiomyopathy, significantly improving the patient’s prognosis [51, 52]. However, careful patient selection and shared decision-making – centred on detailed discussions of procedural risks, potential benefits, and individual health factors – are essential for achieving optimal treatment outcomes.

While catheter ablation is effective, it is not without risks. Procedural complications, although rare, can include cardiac tamponade, thromboembolism, phrenic nerve lesion, atrio-oesophageal fistula and pulmonary vein stenosis [1].

## Emerging therapeutic approaches

The antiarrhythmic drugs that are currently employed to treat AF exhibit limited efficacy and frequently give rise to safety concerns. Moreover, it should be noted that the drugs in this category were not initially developed with AF as their primary target and, consequently, their mechanisms of action are not tailored to target the complex and evolving nature of AF [4, 17, 18]. Therefore, novel atrial-specific targets are currently being investigated with the objective of enhancing the effectiveness of AADs while concurrently reducing the potential risks of ventricular proarrhythmia.

*I*_Kur_ blockers, expected to selectively prolong atrial APD, have largely disappointed in clinical trials, as have *I*_K,ACh_ inhibitors, which have shown limited success in maintaining rhythm control [53]. Research into SK channels is ongoing, with a recent Phase 2 study meeting efficacy and safety endpoints for pharmacologic cardioversion of patients with recent onset AF, with 90-minute cardioversion rates up to 55 % [19]. Despite these promising results, the clinical role of SK channel blockers in AF management remains to be fully established, as early trials reported mild QTc prolongation, and evidence suggests that SK channels may paradoxically facilitate arrhythmogenic triggers in certain settings by promoting triggered activity [4, 19].

The TASK-1 channel contributes to the shortening of action potential duration associated with AF [20, 23]. Additionally, several commonly used antiarrhythmic drugs have demonstrated off-target effects on K_2P_ channels [21]. The identification of a drug that selectively inhibits atrial* I*_TASK 1_-without impacting TASK-1 channels in the central nervous system, pulmonary artery, or adrenal gland could render TASK-1 an optimal target for an atrial-specific AAD [27, 28]. Currently, the DOCTOS study is exploring the use of doxapram, an FDA- and EMA-approved respiratory stimulant that blocks *I*_TASK-1_, for acute cardioversion of paroxysmal and persistent AF [22]. The specificity for atrial myocardium reduces the risk of ventricular proarrhythmia, which is a common limitation of broader ion channel blockers. The focus on TASK-1 inhibition exemplifies a targeted approach in AF management aimed at molecular modulation of atrial ion channel activity, marking a significant step forward in AF pharmacotherapy [4].

**Figure 1:**
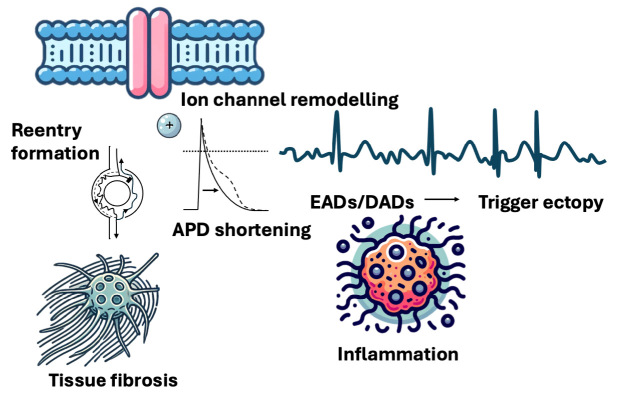
Molecular mechanisms of atrial arrhythmogenesis APD, action potential duration; DADs, delayed afterdepolarizations; EADs, early afterdepolarizations.

Multi-channel blockers like HBI-3000, which target several ion channels simultaneously, are being evaluated for rhythm control without the adverse proarrhythmic effects typically associated with AADs [54]. Another novel agent, HSY244, showed insufficient efficacy in a recent trial, leading to its discontinuation [55].

Additional strategies include alternative drug delivery methods such as intrapericardial administration of AADs, nasal application of flecainide, and botulinum toxin injections [4]. Combination therapies have shown promise as well; for example, the HARMONY trial demonstrated that combining ranolazine and low dose dronedarone effectively reduced AF burden [56].

Upstream therapies targeting inflammation and fibrosis are also under investigation, as these factors contribute to AF’s progression. Canakinumab, targeting IL-1β, and metformin have shown potential, though further clinical trials are required [17, 18]. Further, colchicine and SGLT2 inhibitors are being evaluated for their anti-inflammatory and antifibrotic effects on AF management [57].

Finally, lifestyle and risk factor management are integral to reducing AF burden, with pharmacotherapy being explored in support. Drugs such as antihypertensives, weight-loss agents, and omega-3 supplements are under review for their potential to lower AF risk [4]. Ultimately, a more targeted approach to distinct AF subtypes may reveal better uses for AADs in future treatment plans.

## Conclusion

In conclusion, advancing insights into the molecular mechanisms underlying AF have highlighted novel targets for more precise and effective therapeutic interventions. Key pathways, including ion channel dysregulation – such as for example TASK-1 potassium channels – calcium mishandling, oxidative stress, and fibrosis, drive AF pathophysiology and sustain arrhythmogenic conditions. Emerging therapies focusing on these pathways promise to enhance AF management beyond traditional rate and rhythm control, potentially reducing recurrence and improving patient outcomes. Tailoring treatment to address the individual cellular and molecular bases of AF may represent the future of durable and patient-centred arrhythmia care.
